# Differences in Ear Rot Resistance and *Fusarium verticillioides*-Produced Fumonisin Contamination Between Polish Currently and Historically Used Maize Inbred Lines

**DOI:** 10.3389/fmicb.2019.00449

**Published:** 2019-03-18

**Authors:** Elżbieta Czembor, Agnieszka Waśkiewicz, Urszula Piechota, Marta Puchta, Jerzy H. Czembor, Łukasz Stȩpień

**Affiliations:** ^1^Department of Grasses, Legumes and Energy Plants, Plant Breeding and Acclimatization Institute – NRI, Radzikow, Blonie, Poland; ^2^Department of Chemistry, Poznań University of Life Sciences, Poznań, Poland; ^3^National Centre for Genetic Resources, Plant Breeding and Acclimatization Institute – NRI, Radzikow, Blonie, Poland; ^4^Department of Pathogen Genetics and Plant Resistance, Institute of Plant Genetics, Polish Academy of Sciences, Poznań, Poland

**Keywords:** maize inbred lines, *Fusarium* ear rot, fumonisin accumulation, genetic distance assessment, ddRADseq

## Abstract

Poland is the fifth largest European country, in terms of maize production. Ear rots caused by *Fusarium* spp. are significant diseases affecting yield and causing grain mycotoxin contamination. Inbred lines, which are commonly used in Polish breeding programs, belong, mostly, to two distinct genetic categories: flint and dent. However, historically used lines belonging to the heterotic Lancaster, IDT and SSS groups were also present in previous Polish breeding programs. In the current study, 98 inbred lines were evaluated across a 2-year-long experiment, after inoculation with *F. verticillioides* and under natural infection conditions. Lancaster, IDT, SSS and SSS/IDT groups were characterized as the most susceptible ones and flint as the more resistant. Based on the results obtained, the moderately resistant and most susceptible genotypes were defined to determine the content of fumonisins (FBs) in kernel and cob fractions using the HPLC method. Fumonisin's content was higher in the grain samples collected from inoculated plants than in cobs. The association of visible *Fusarium* symptoms with fumonisin concentration in grain samples was significant. Conversely, the cobs contained more FB_1_ under natural infection, which may be related to a pathogen's type of growth, infection time or presence of competitive species. Using ddRADseq genome sampling method it was possible to distinguish a basal relationship between moderately resistant and susceptible genotypes. Genetic distance between maize genotypes was high. Moderately resistant inbreed lines, which belong to IDT and IDT/SSS belong to one haplotype. Genotypes which belong to the flint, dent or Lancaster group, and were characterized as moderately resistant were classified separately as the same susceptible one. This research has demonstrated that currently grown Polish inbred lines, as well the ones used in the past are a valid source of resistance to *Fusarium* ear rot. A strong association was observed between visible *Fusarium* symptoms with fumonisin concentration in grain samples, suggesting that selection in maize for reduced visible molds should reduce the risk of mycotoxin contamination. NGS techniques provide new tools for overcoming the long selection process and increase the breeding efficiency.

## Introduction

Maize has become one of the most important crops for food and feed production worldwide–both as silage and crop residue. It is also used industrially for starch and oil extraction. The production area in Poland has reached 1200 thousand ha. Fungal diseases are among the most important factors limiting the yield and grain quality of maize, with *Fusarium* ear rots (FER) caused by *Fusarium* at the top of the list. *Fusaria* has the ability to produce toxins which are harmful to both humans and animals alike. The environment has a huge impact on these processes and this is also being studied. In Europe the most common ear rotting fungal species are: *F. verticillioides* (Sacc.) Nirenberg, causing pink ear rot, and *F. graminearium* Schwabe, causing red ear rot. However, a number of minor species are also present: *F. temperatum, F. poae, F. proliferatum, F. avenaceum, F. culmorum F. subglutinans*, and *F. sporotrichioides*. Maize grain is mostly contaminated with fumonisins produced by *F. verticillioides* and *F. proliferatum*, and/or by deoxynivalenol and other trichothecenes along with zearalenone produced by *F. graminearum*, which affect the health of human and animals (Desjardins and Plattner, 2000; Logrieco et al., 2002; Bennett and Klich, 2003; Munkvold, 2003b; Oldenburg and Ellner, 2005; Voss et al., 2006; Dorn et al., 2009; Czembor et al., 2014, 2015; Gallo et al., 2015; Stoycho, 2015; Miedaner et al., 2017). The maximum acceptable levels of mycotoxin content have been established for both maize and maize products. The guidance has been placed for foodstuffs (EC, 2007) and for animal feed in the European Union (EC, 2006). Contamination of maize grain or feed product is influenced by environmental conditions, agricultural practices, genotype resistance and the interaction between all of these factors (Maiorano et al., 2009a,b; Vasileiadis et al., 2011; Zijlstra et al., 2011; Mesterhazy et al., 2012; Cao et al., 2014b; Miedaner et al., 2017). *Fusarium verticillioides* can cause disease, at all developmental stages of the plant, in some cases without displaying any symptoms and, consequently, fumonisins are present in symptomless infected kernels (Desjardins and Plattner, 2000; Desjardins et al., 2002; Miedaner et al., 2010).

Ear rot caused by *F. verticillioides* favored warm and dry conditions, however, warm and wet conditions following silking have been reported to be conducive for disease development (Munkvold, 2003b). Weather conditions during flowering are critical for primary infection, as well as for toxin accumulation during flowering and then before harvesting (De La Campa et al., 2005; Maiorano et al., 2009a,b; Cao et al., 2014b). Low rainfall and a high number of days with maximum temperatures around 30-35°C during flowering favor disease development. Additionally, precipitation stimulates mycotoxin accumulation before the maturity stage because of the extended harvest period.

Appropriate agronomic practice is one of the most effective approaches to reduce mycotoxin contamination of maize grain (Munkvold, 2003a; Ariño et al., 2009; Mesterhazy et al., 2012; de Galarreta et al., 2015) along with the use of less susceptible hybrids, which can be developed either by traditional breeding methods or by transgenic technology (Munkvold, 2003a; Smith et al., 2004; Presello et al., 2005, 2011; Butrón et al., 2006; Smith, 2007; Toldi et al., 2008; Eller et al., 2010; Lanubile et al., 2011; Mesterhazy et al., 2012; Czembor et al., 2015). Genetic resistance is needed in currently used cultivars and it can be deployed from available intra-specific variability. The understanding of the mechanisms underlying maize resistance to ear rot is still limited. Their nature is polygenic and mapped resistance quantitative trait loci (QTL) have relatively small effects and are not consistent between populations (Pérez-Brito et al., 2001; Robertson-Hoyt et al., 2006; Ding et al., 2008; Xiang et al., 2010; Ali and Yan, 2012; Chen et al., 2012; Yuan et al., 2013; Zila et al., 2013, 2014; Mideros et al., 2014; Butrón et al., 2015; Lanubile et al., 2017). The accumulation of mycotoxins can also be affected by the plant genotype. Pedigree breeding has caused maize inbreeds to become not only more-elite but also genetically more uniform. Older generations of inbred lines are still used in inbred line development and genetic studies or as testers in many breeding programs (Presello et al., 2005, 2011; Mesterhazy et al., 2012). The most utilized inbreds belong into the heterotic groups such as Reid Yellow Dent, Iowa Stiff Stalk Synthetic (SSS), Lancaster, Iodent (IDT) and have not been subjected to phenotypic selection for FER resistance. Flint and popcorn hybrids tend to exhibit less FER severity and grain mycotoxin contamination than dent ones, though resistant dent hybrids are also available (Presello et al., 2007). The stability of the earlier germplasms from Argentina was evaluated in Argentinian and Canadian environments (Presello et al., 2004, 2005, 2011). German conditions were evaluated by Bolduan et al. (2009) and Löffler et al. (2010) who concluded that the sources of resistance are effective in different locations. South African conditions were evaluated by Small et al. (2012), Italian by Balconi et al. (2014) Central European (Poland) by Czembor and Frasinski (2018) and Czembor et al. (2013a,b, 2015).

The breeding programs intensified the development of more resistant genotypes and the identification of new resistance sources, the plant selection procedures and QTL mapping. The resistance mechanism is also being studied. Recently, next-generation sequencing (NGS) technology has emerged as a cutting-edge approach for high-throughput sequence determination. It has improved the efficiency and speed of gene discovery, reducing the time, labor, and cost (Robertson et al., 2006; Elshire et al., 2011; Mesterhazy et al., 2012; Peterson et al., 2012; Zila et al., 2014).

In summary the objectives of this study were: (i) to evaluate the variation of FER resistance and mycotoxin contamination caused by *F. verticilliides* among a broad base of early, mid-early and late groups of maize elite inbred lines belonging to currently used flint and dent groups as well as historical heterotic groups such as Lancaster, IDT, SSS, and (ii) to estimate the level of genetic diversity among and within these groups using NGS technology. Inbreds possessing resistance to FER and fumonisin accumulation would be valuable materials for future breeding programs.

## Materials and Methods

### Plant Materials

**Figure 1:**
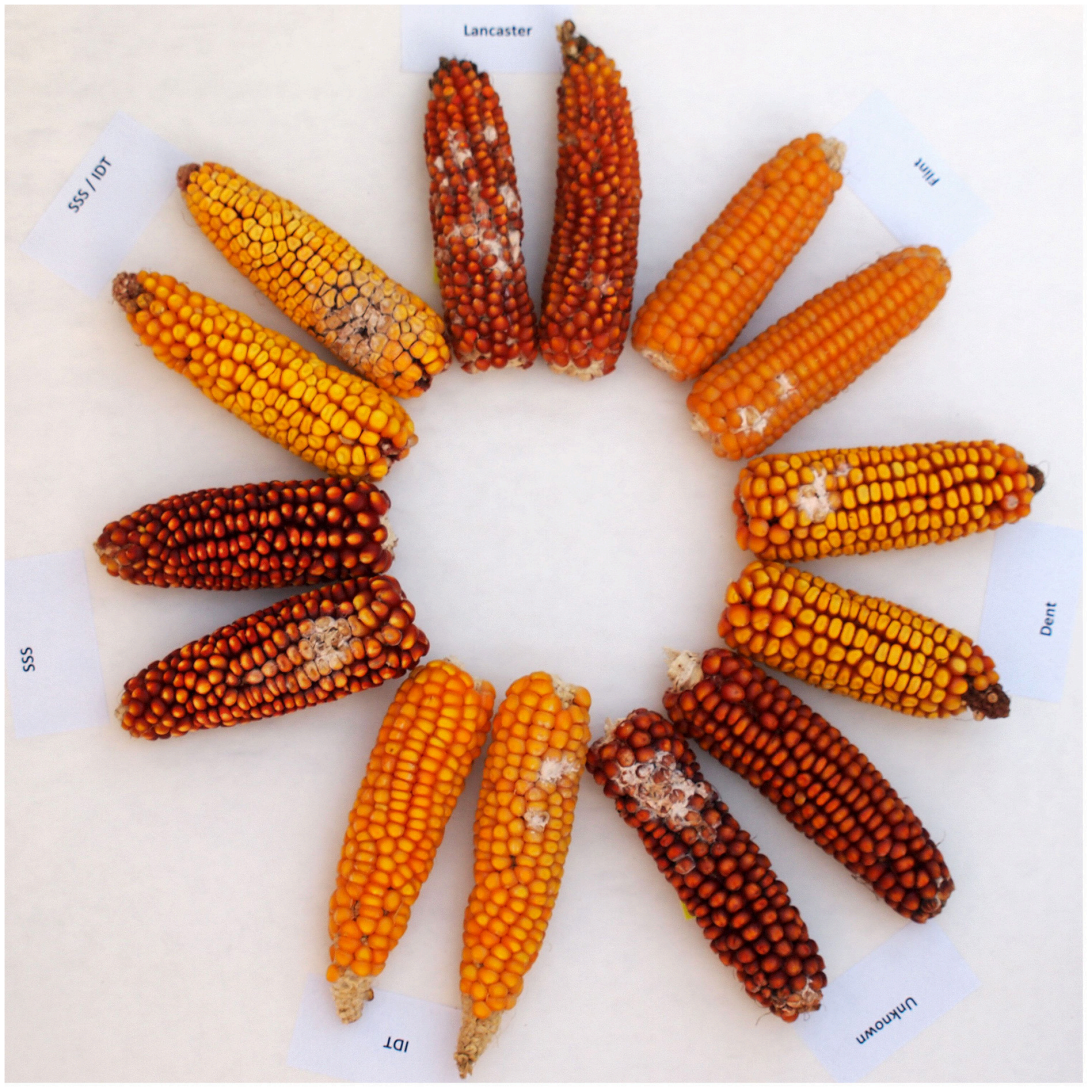
Ears morphology of inbred lines belonging to different heterotic groups: flint, dent, Lancaster, Iodent (IDT), Stiff Stalk Synthetic (SSS), SSS/IDT and unknown groups and symptoms of the *Fusarium* ear rot (FER) disease after kernel inoculation with *F. verticillioides*.

Ninety-eight inbred lines were evaluated, belonging to currently used flint (*n* = 23) and dent (*n* = 39) inbred groups, as well as historically used heterotic groups such as Lancaster (*n* = 4), Iodent Reid (IDT, *n* = 9), Stiff Stalk Synthetic (SSS, *n* = 8) and SSS/IDT (S/I, *n* = 5).A heterotic group of 10 lines was unknown. Based on phenotypic FER assessment, genotypes were divided into 4 groups: highly resistant, moderately resistant, moderately susceptible and very susceptible. Moderately resistant and very susceptible genotypes, representing each heterotic group (30 inbreds: 10 dents, 8 flints, 3 IDT, 2 Lancaster, 3 SSS, 2 SSS/IDT, 2 of the unknown heterotic group), were selected for further research aimed at determining the content of fumonisins in grain and cob fractions (Figure 1).

### Field Experiment and Phenotypic Ear Rot Resistance Assessment

Two-year field experiments were conducted in Radzików, Central Poland (N: 52.21929, 20.63137, sea level: 87 m), across 2011-2012 seasons, as was suggested by Bolduan et al. (2009). They recommended a two-stage selection: an online personal contribution by artificial infection at one place and the next between test hybrids in several (2–3) locations. An RCBD (randomized complete block design) model was used for the field experiment. About 25 plants of maize inbred lines were grown in one row, in three replications (0.75 m between rows and 0.25 m between plants in the row).

To produce inoculum, a well-characterized fumonisin-producing *F. verticillioides* strain was used. This isolate was selected from the collection carried out by Plant Breeding and Acclimatization Institute-NRI based on the results of a 2-year experiment as the most aggressive in relation to two hybrids of maize. A PDA (Potato dextrose agar) plug with 7-day-old culture was transferred to a 10-mL vial containing 12 autoclaved toothpicks and 8-mL SNA (Synthetic low-Nutrient Agar) and incubated for 2 weeks at 25°C. Prior to autoclaving, the toothpicks were washed and boiled in excess water for 1 h to remove any toxic substances that might inhibit fungal growth (Jardine and Leslie, 1992). After incubation the toothpicks were removed from the vials and air-dried on a sterile bench overnight.

Inoculation of individual ears was conducted 10–12 days after silking time. At least 20 plants were inoculated for each genotype (6–7 plants in three replicates). Control plants were inoculated using toothpicks without the pathogen. At maturity, ears from each plot were dehusked and harvested manually, dried to approximately 15% grain moisture content and individually rated for FER symptoms using a seven-point scale: 1 = no visible disease symptoms, 2 = 1–3%, 3 = 4-10%, 4 = 11–25%, 5 = 26–50%, 6 = 51–75%, and 7 = 76–100% of kernels exhibiting visual symptoms of infection, such as brown, pink, or reddish discoloration of kernels and pinkish or white mycelial growth (Clements et al., 2003a,b; Supplementary Figure 1). Ear rot severity scores were converted to percentages of the ear exhibiting symptoms replacing each score with the mid-point of the interval.

### Fumonisins Analysis

Fumonisin B_1_, B_2_, B_3_ standards were purchased with a standard grade certificate from Sigma-Aldrich (Steinheim, Germany). Sodium dihydrophosphate, potassium chloride, acetic acid and *o*-phosphoric acid were purchased from POCh (Gliwice, Poland). Organic solvents (HPLC grade), disodium tetraborate, 2-mercaptoethanol and all the other chemicals were also purchased from Sigma-Aldrich (Steinheim, Germany). Water for the HPLC mobile phase was purified using a Milli-Q system (Millipore, Bedford, MA, USA).

Extraction and purification procedure: 10 g of homogenized ground samples of maize kernels were prepared for analysis. Mycotoxins (FBs) were extracted and purified according to the detailed procedure described earlier (Waśkiewicz et al., 2013a,b). The eluates were evaporated to dryness at 40°C under a stream of nitrogen. The dry residue was stored at −20°C until the HPLC analyses. The chromatographic system consisted of Waters 2695 high-performance liquid chromatography (Waters, Milford, USA) with Waters 2475 Multi λ Fluorescence Detector (λex = 335 nm, λem = 440 nm) with an XBridge column (3.0 × 100 mm). Quantification of mycotoxins was performed by measuring the peak areas at the retention time according to a relevant calibration curve. The limit of detection was 0.01 μg g^−1^.

### Genetic Distance Assessment

The level of genetic diversity among 17 distinct maize genotypes was analyzed using NGS technology. They were selected as moderately resistant and susceptible to ear rot, based on the evaluation under field conditions after inoculation and fumonisin content.

For different combinations of restriction enzymes, it is possible to perform sequencing from 0.01 to 1.99% of maize genome size due to the ddRAD *in silico* pipeline (Yang et al., 2016). That fraction selected by the ddRADseq pipeline is enough for a broad spectrum of research. The ddRADseq method is used in phylogeny analysis and establishing a genetic relationship among different genotypes. Different restriction enzymes and broad or narrow size selection make the library proper to MiSeq capacity. DdRADseq does not need a reference sequence (Peterson et al., 2012).

Genomic DNA was extracted from 200 mg of 14-day-old single plant leaf tissue by CTAB method (Waśkiewicz et al., 2013b) with RNase A (Qiagen) treatment before precipitation (30 min at 37°C). DNA quality was evaluated on 1% agarose gel and quantity was measured by NanoDrop 1000 (NanoDrop Technologies Inc., USA).

Double-digest restriction-associated DNA sequencing (ddRADseq) protocol described previously by Peterson et al. (2012) was used to detected molecular data. The library was prepared using 200 ng genomic DNA of each sample. DNA was digested at 37°C for 3 h using the 8U HindIII and 8U FspBI restriction enzymes. In the next step, digested DNA was ligated to adapters using T4 Ligase for 2 h at 16°C. Adapters included Illumina primer sequences and unique barcodes. Purification of ligation products was prepared by AMPure (Beckman Coulter, USA) with beads: sample ratio 1:1. Cleaned DNA was re-suspended in 150 μl of water. Fifteen microlite of ligation products were amplified with NeBNext Ultra II Q5 Master Mix (New England Biolabs, UK) using primers with Illumina index sequences due to the single-indexing method. PCR programme: preliminary denaturation 98°C/30 s., 16 cycles of denaturation at 98°C/10 s. and combined annealing and elongation 65°C/75 s., final elongation 65°C/5 min., hold at 4°C. All PCR products were pooled and cleaned-up with AMPure (Beckman Coulter, USA) with bead: sample ratio 1:1 and next 0.6: 1 to size selection of 400-600 bp. A library was quantified on fluorimeter Qubit (ThermoFisher Scientific, USA) by Quant-iT™ High-Sensitivity dsDNA Assay Kit. A library was diluted to 4 nM and denatured with 0.2N NaOH for 5 min. A denatured library was diluted to 20 pM. 600 μl of a prepared library was sequenced using Illumina MiSeq with Miseq Reagent Kit v3 and final PhiX concentration of 12.5 pM and pair reading protocol for 151 cycles.

Quality control of reads was checked in FastQC software (https://www.bioinformatics.babraham.ac.uk/projects/fastqc/; FastQC A Quality Control tool for High Throughput Sequence Data by: S. Andrews). Reads-analysis were prepared with Geneious 11.0 software (https://www.geneious.com, Kearse et al., 2012). The parameters of the substitution model define a rate matrix that can be used to calculate the probability of evolving from one base to another in a given period of time. Most models are variations of two sets of parameters–the equilibrium frequencies and relative substitution rates. Genetic Distance Model: Tamura-Nei. This model also assumes different equilibrium base frequencies. In addition to distinguishing between transitions and transversions, it also allows the two types of transitions (A ↔ G and C ↔ T) to have different rates (Tamura and Nei, 1993). Before analyzing, the reads were split by barcode data. Then, the ends were trimmed, paired reads merged and duplicate reads removed (Supplementary Figure 2). The reads were *de novo* assembled using original data by the Geneious *de novo* overlap assembler to a high accuracy level (Supplementary Figure 3). A genotype tree was made using the neighbor-joining method with a genetic distance model. The tree was constructed with standard software settings. In this method, neighbors are defined as a pair of leaves with one node connecting them, minimizing the total branch length at each stage of clustering, starting with a star-like tree. The branch lengths and an unrooted tree topology can quickly be obtained by using this method without assuming a molecular clock (Saitou and Nei, 1987).

### Meteorological Data

**Figure 2:**
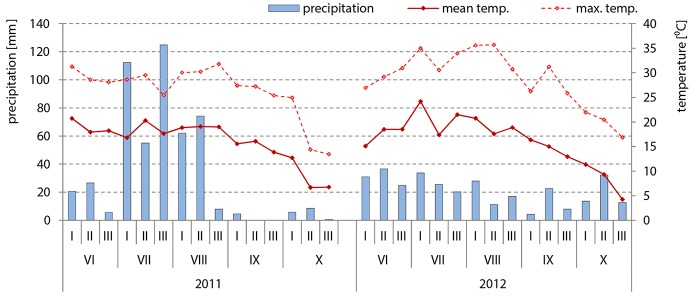
Weather conditions in the years 2011 and 2012 when the FER resistance assessment of 98 maize inbred lines was carried out under field conditions with a natural infection and after inoculation with *F. verticillioides*: mean temperature and precipitation data from sowing time (April) till harvesting time (October) were shown.

Field trials were conducted in Radzików, Central Poland. The generative stages of hybrids belonging to the middle-early group were as follows: silking (R1)–I decade of July, blister (R2)–II decade of July, milk (R3)–III decade of July, dough (R4)–I decade of August, dent (R5)–from the II decade of August until the end of I decade of September. Physiological maturity (R6) stage and harvesting time were in the II decade of September. The generative stages of hybrids belonging to the middle-late group started 7 days later (II decade of July) and lasted until the half of the III decade of September. The average temperatures and rainfalls during this time are presented in Figure 2.

### Statistical Analyses

Comparison between inbred lines was done using Fisher's least significant difference test using InfoStat software. In addition to the mold-covered surface proportion of the maize grain, fumonisin content in the infected maize grain meal and the content of the infected maize grain meal, the so-called Toxin-Mold-Index (TMI), was also calculated according to the method described by Arpad et al. (1997), by multiplying the sum of mycotoxin contents, expressed in mg per kg, by the proportion of mold-covered surfaces of maize ear expressed as percentage of the total surface where: TMI = (FB_1_+FB_2_+FB_3_)^*^A; FB_1_, FB_2_, FB_3_ content in maize meal obtained from grain and corn cobs; A - proportion of the mold-covered area of ears in % of the total areas of ears.

Differences between inbred lines within years were determined with Fisher's protected least significant difference (LSD) test. Linear correlation coefficients were determined for the relationship between FER severity and fumonisin concentration in grain and cobs sampled from non-inoculated and inoculated plants in 2012 using InfoStat software.

## Results

### *Fusarium* Ear Rot Severity and Fumonisin Contamination

All inoculations resulted in visible disease symptoms (DI = 100%). A summary of statistical data such as mean, SD, minimum and maximum of ear rot ratings of flint (*n* = 23), dent (*n* = 39), IDT (*n* = 9), Lancaster (*n* = 4), SSS (*n* = 8), SSS/IDT (*n* = 5), and unknown (*n* = 10) heterotic groups under natural infection (non-inoculated) and inoculated with *F. verticillioides* in 2011 and 2012 are shown in Table 1. The Variability of ear rot incidence was observed between, and within, heterotic groups. Figure 1 shows ears representing individual origin groups and symptoms of the disease after inoculation.

**Table 1:** Summary statistics of ear rot ratings of *n* = 98 inbred lines that belong to flint, dent, Lancaster, SSS, IDT, SSS/IDT and “unknown” heterotic groups under natural infection and inoculated with *F. verticillioides* in 2011 and 2012 years.

**No**.	**Heterotic group**	**n**	***Fusarium*** **ear rot (%)**							
			**non-inoculated**				**inoculated**			
			**2011**		**2012**		**2011**		**2012**	
			**mean^**a**^**	**range**	**mean^**a**^**	**range**	**mean^**a**^**	**range**	**mean^**a**^**	**range**
1	dent	39	2.2 ± 0.62	0.0–7.0	5.4 ± 6.76	1.2–48.0	6.8 ± 4.35	1.2–22.0	14.9 ± 11.75	2.0–63.0
2	flint	23	1.4 ± 0.56	0.0–4.5	4.3 ± 3.47	1.6–45.5	9.1 ± 6.85	2.0–45.5	16.4 ± 9.35	3.0–58.0
3	IDT	9	1.8 ± 0.80	0.0–4.5	3.4 ± 2.23	1.6–18.0	6.3 ± 4.27	1.2–18.0	11.3 ± 8.06	4.0–34.0
4	lancaster	4	1.9 ± 0.75	0.0–7.0	8.6 ± 7.53	1.2–22.0	16.3 ± 17.89	2.0–50.5	10.9 ± 7.54	5.0–22.0
5	SSS	8	1.8 ± 0.78	0.0–7.0	4.5 ± 5.54	1.6–26.0	6.8 ± 5.20	1.4–15.8	14.7 ± 12.89	3.0–43.0
6	SSS / IDT	5	1.3 ± 0.60	0.0–2.0	7.5 ± 6.50	1.6−22.0	21.7 ± 8.47	7.0–45.5	31.0 ± 16.55	11.4–63.0
7	unknown	10	1.8 ± 0.97	0.0–7.0	3.5 ± 2.10	1.6–18.0	4.2 ± 2.32	1.3–9.2	11.2 ± 5.01	3.0–26.0
Mean		98	4.93 ± 6.13	1.6–45.5	1.82 ± 1.13	0.0–7.0	8.07 ± 7.77	1.4–50.5	15.15 ± 11.55	3.0–63.0
LSD Fisher _p < 0.05_		98	5.941		1.709		7.845		6.319	

a *Mean ear rot severity is reported as the average of the entry least square means (back-transformed to the original 0-100% disease severity scale)*.

In July, when the primary infection usually takes place, maximum temperatures were higher in 2012 than in 2011 by ca. 5°C and favored the development of *F. verticillioides*, used for artificial infection (Figure 2). In 2011, high precipitation in July (112 mm, 55 mm and 127 mm in the first, second and third decades, respectively) and August (62 mm and 74 mm in the first and second decades, respectively) did not stimulate disease development. The effects of maize heterotic groups and inbred lines on disease severity differed between years.

Significant differences in FER severity were observed among inbred lines and heterotic groups evaluated under natural infection and after inoculation in both years. *Fusarium* ear rot severity was significantly higher in 2012 than in 2011 with means of 4.9 and 1.8% of the ear exhibiting disease symptoms under natural conditions, respectively, and 15.2 and 8.07% after inoculation with *F. verticillioides*. Under natural infection, FER severity for all 98 inbred lines ranged from 1.6 to 45.5% of the ear exhibiting disease symptoms in 2012 and from 0.0 to 7.0% of the ear in 2011. After inoculation, FER severity ranged from 3.0 to 63.0% of the ear exhibiting disease symptoms in 2012 and from 1.4 to 50.5% of the ear in 2011. In 2012 the means of ear rot severity of evaluated groups ranged from 10.9 to 30.99% of ears covered by disease symptoms. The most susceptible group was SSS/IDT (Table 1). The most resistant inbred lines belonged, mostly, to the unknown heterotic group. The SD in all heterotic groups ranged from 1.13 to 2.2 and from 2.10 to 6.76 under natural infection in 2011 and 2012, respectively. They were much higher after artificial inoculation; ranging from 2.32 to 17.89 in 2011 and 2012, respectively.

**Figure 3:**
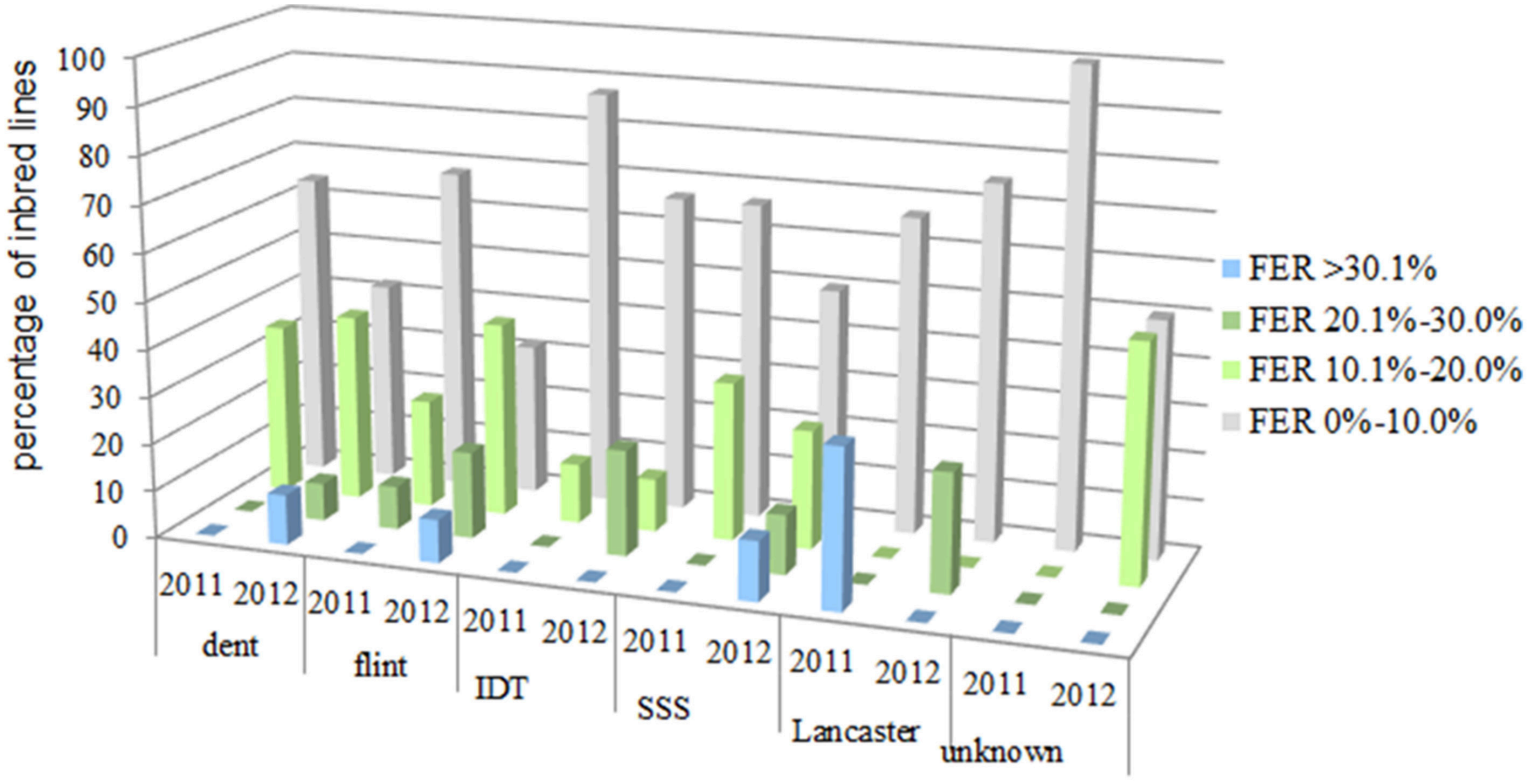
Distribution for percentage data of inbred lines for which the disease symptoms covering: 0.0–10.0%, 10.1–20.0%, 20.1–30.0% and above 30.1% of the total ear area after kernel inoculation with *F. verticillioides* in 2011 and 2012 belonging to different heterotic groups: 23 flint, 39 dent, 4 Lancaster, 9 Iodent (IDT), 8 Stiff Stalk Synthetic (SSS) 5 SSS/IDT and 10 unknown heterogenic group.

On average, for more than 91% of all evaluated inbred lines, the symptoms of the disease did not exceed 10% of the total ear area under natural infection (Figure 3). In 2011, for 73% of all evaluated inbred lines, the symptoms of the disease covered <10% of the ear area, and in 2012 the same applied to 43.9% of lines tested. In 2012, for 10.2% of evaluated inbred lines, the disease symptoms were observed within a range of 26–50% of ear area.

**Figure 4:**
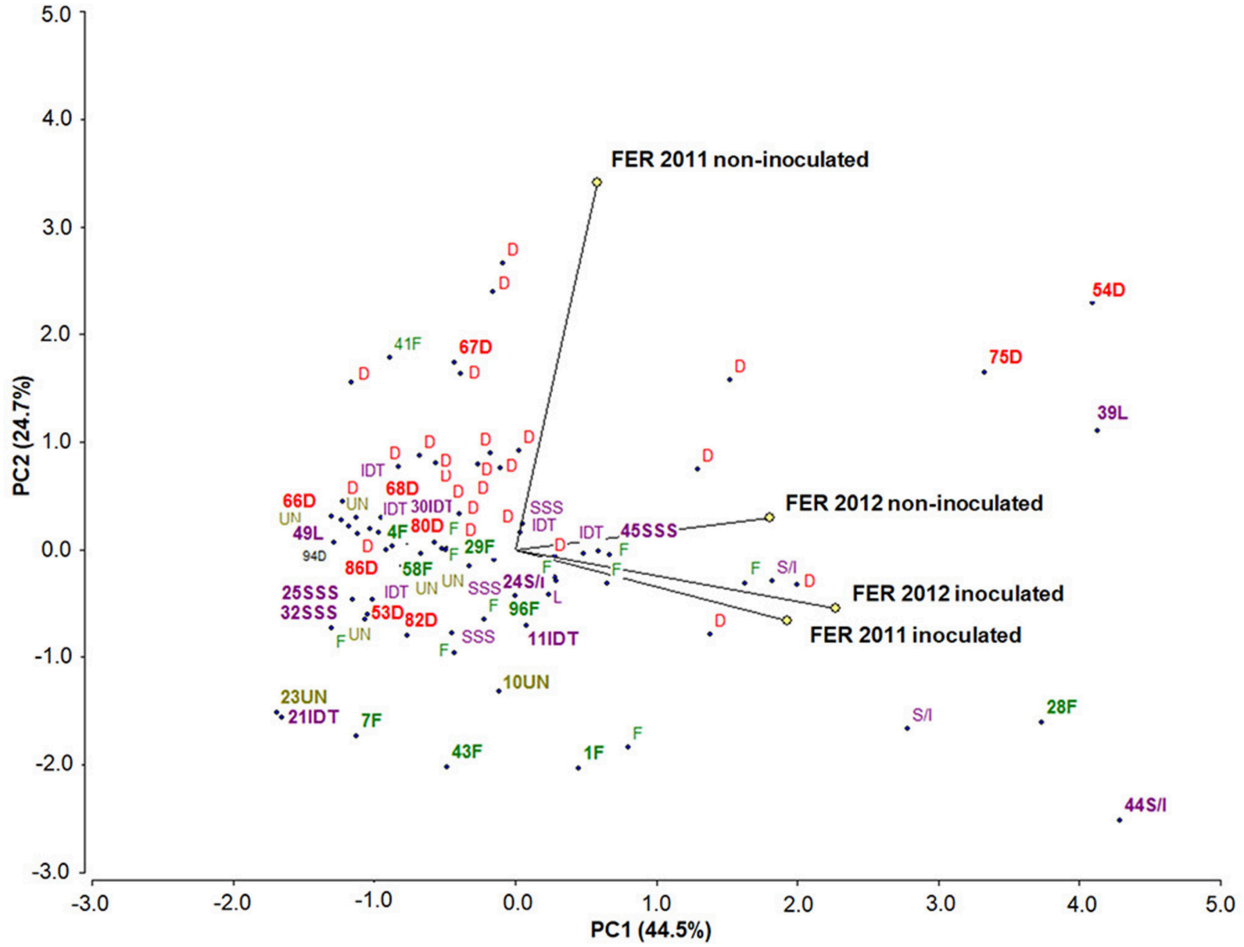
Scatter plot of the first principal component scores derived from rating the FER severity, showing the dispersion of 98 inbred lines belonging to different heterotic groups: 23 flint (F), 39 dent (D), 4 Lancaster (L), 9 Iodent (IDT), 8 Stiff Stalk Synthetic (SSS), 5 SSS/IDT and 10 unknown (UN). Colored dots represent individual subjects with a subject identification group. Colored dots represent individual subjects with a subject identification number were selected for fumonisin content analysis as a moderate resistant (negatively correlated with FER) and susceptible one (positively correlated with FER). The length of the vectors represents the PCA loadings of the variables on the first two principal components, which explain 69.2% of the variability.

A simple correlation analysis and a principal component analysis (PCA, Figure 4) were performed to decipher the relationships between FER severity under natural infection and after inoculation. Using Pearson's correlation coefficients it was shown that the FER severity under natural infection in 2012 was significantly correlated with FER after the *F. verticillioides* kernel inoculation (*r* = 0.48^*^, *p* < 0.005). Furthermore, the results of FER scored in 2011 after inoculation was positively correlated with the results obtained in 2012 (0.46^*^, *p* < 0.005). The purpose of the PCA was to group individual parameters in a more comprehensive manner. Two principal components (PC) explained 69.2% of the variability. The length of the vectors represents the PCA loadings of the variables on the first two principal components, PC1 (44.5%) had strong positive loading from FER after inoculation in 2011 and 2012 and FER under natural infection in 2012. PC2 (24.7%) had positive loading from FER under natural infection in 2011. The highest diversity among inbreds was associated with FER, evaluated after inoculation in 2012. The most susceptible and moderately resistant inbred lines from each group of origin were selected for fumonisin content analysis.

**Figure 5:**
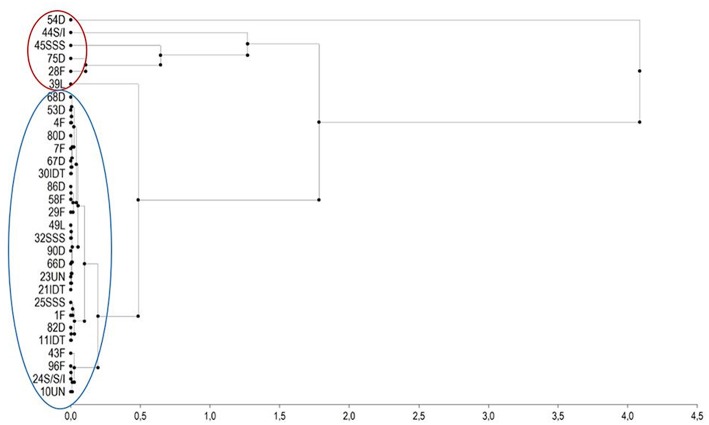
Hierarchical clustering of the inbred lines representing moderately resistant and very susceptible genotypes selected from the collection of 98 inbred based on the disease assessment of non-inoculated and inoculated ears with *F. verticillioides* in 2011 and 2012 years using the Euclidean distance of the elements of relationship matrix A (based on TMI index genotypes in a red cluster represent a very susceptible group). Thirty inbred lines belonging to different heterotic groups 10 dent (D), 8 flint (F), 3 Iodent (IDT), 2 Lancaster (L), 3 Stiff Stalk Synthetic (SSS), 2 SSS/IDT, and 2 unknown (UN). Red color circle: susceptible group, blue color circle: moderate resistant group.

Ear rot severity of these genotypes in 2011 and 2012, fumonisin contamination in grain and cobs sampled from inoculated and non-inoculated plants in 2012 and TMI index are shown in Table 2. Under natural infection, cobs were more contaminated with fumonisin B_1_ than grain samples. The frequency of cob samples contaminated with FB_1_ was 66.7% and for grain samples contaminated with FB_1_ it was 29.6%. Maize inbred lines accumulated different levels of fumonisins in grain and in cobs. In grains sampled from inbred lines of dent, flint, SSS, IDT, SSS/IDT and Lancaster heterotic groups after kernel inoculation with *F. verticillioides*, the FB_1_ contamination ranged from 0.99 to 99.04 mg/kg, FB_2_ contamination ranged from 0.05 to 21.44 mg/kg and FB_3_ from 0.0 to 2.21 mg/kg. In cob samples FB_1_ contamination ranged from 0.02 to 23.05 mg/kg, FB_2_ from 0.0 to 4.5 mg/kg and FB_3_ from 0.0 to 1.82 mg/kg. Based on the TMI index it was possible to identify highly resistant and susceptible genotypes. A hierarchical clustering of the inbred lines representing resistant and very susceptible genotypes belonging to each heterotic group was created based on the TMI index (Figure 5). Inbreds with low fumonisin content belonged to two separate clusters with low distance between them. Susceptible inbreds belonged to the very inconsistent group with large distances from each other.

**Table 2:** *Fusarium* ear rot resistance (FER) of elite inbred lines under natural infection and after *Fusarium verticillioides* inoculation, fumonisin content in grain and cobs sampled in 2012 along with the Toxin-Mold-Index (TMI).

**Heterotic group**	**Inbred line**	**FER**		**Toxin content**								**TMI**	
		**non-inoculated**	**inoculated**	**non-inoculated**		**inoculated**							
				**grain**	**cobs**	**grain**			**cobs**				
				**FB_**1**_**	**FB_**1**_**	**FB_**1**_**	**FB_**2**_**	**FB_**3**_**	**FB_**1**_**	**FB_**2**_**	**FB_**3**_**	**non-inoculated**	**inoculated**
dent (*n* = 10)	53D	2.3	8.1	0.00	0.21	5.35	0.93	0.04	4.36	0.26	0.04		
	54D	10.3	56.3	0.00	0.39	99.04	21.44	2.21	6.50	1.65	0.29	4.0	7378.5
	66D	2.9	2.7	0.00	0.00	5.04	0.58	0.04	0.17	0.06	0.01	0.0	15.5
	67D	4.3	7.5	0.00	0.00	5.41	0.33	0.05	3.31	0.16	0.03	0.0	66.3
	68D	2.1	10.7	0.00	0.00	7.70	0.57	0.05	1.60	0.33	0.04	0.0	106.4
	75D	26.5	39.7	1.07	0.88	44.74	8.41	0.67	1.10	0.25	0.03	51.8	2174.3
	80D	2.3	7.0	0.00	0.00	7.57	1.08	0.12	1.71	0.56	0.07	0.0	77.8
	82D	37.3	12.1	0.00	0.00	12.43	1.22	0.07	2.84	0.87	0.14	0.0	211.7
	86D	2.5	11.4	0.00	0.61	10.31	0.98	0.11	0.85	0.22	0.01	1.6	137.5
	90D	2.9	5.2	0.31	0.00	3.69	0.28	0.05	0.08	0.01	0.00	0.9	21.4
	Mean	9.4 ± 7.23	16.1 ± 11.96	0.14 ± 0.33	0.21 ± 0.31	20.13 ± 29.73	3.58 ± 6.57	0.35 ± 0.71	2.25 ± 2.03	0.44 ± 0.49	0.07 ± 0.09	5.9 ± 17.14	1026.0 ± 2275.4
flint (*n* = 8)	1F	3.5	16.5	0.00	0.40	5.67	1.73	0.21	5.39	1.22	0.36	1.4	241.3
	28F	18.8	40.3	0.00	0.19	45.75	9.95	1.03	0.02	0.00	0.00	3.4	2351.2
	29F	4.0	16.3	0.00	0.00	6.46	4.33	0.11	0.14	0.05	0.01	0.0	161.3
	43F	2.5	7.7	0.20	0.20	26.14	3.85	0.40	23.05	4.01	1.82	1.0	454.0
	4F	5.2	8.1	1.06	0.00	7.38	1.10	0.15	2.62	0.61	0.25	5.5	99.3
	58F	2.3	9.9	0.00	0.42	7.03	0.96	0.14	4.46	0.75	0.04	1.0	134.3
	7F	5.0	8.1	0.00	0.00	5.32	0.72	0.11	0.17	0.06	0.01	0.0	52.0
	96F	3.9	17.9	0.00	0.11	14.40	2.15	0.14	5.14	1.32	0.29	0.4	409.5
	Mean	5.7 ± 5.33	15.6 ± 10.19	0.16 ± 0.36	0.17 ± 0.16	14.77 ± 14.47	3.10 ± 3.31	0.29 ± 0.28	5.12 ± 7.33	1.00 ± 1.27	0.35 ± 0.59	1.58 ± 2.21	487.9 ± 792.37
IDT (*nn* = 3)	11IDT	3.2	23.3	0.00	0.00	3.37	0.29	0.05	3.90	0.83	0.30	0.0	207.6
	21IDT	1.7	6.4	0.00	0.00	0.98	0.05	0.00	0.27	0.03	0.00	0.0	9.0
	30IDT	2.6	6.3	0.00	0.00	7.44	1.11	0.11	1.01	0.10	0.02	0.0	61.8
Lancaster (*n* = 2)	39L	17.1	22.0	0.00	0.41	15.41	2.08	0.29	19.76	4.50	1.43	7.0	956.3
	49L	3.3	6.0	0.00	0.00	3.38	0.30	0.03	0.08	0.01	0.00	0.0	22.8
SSS (*n* = 3)	25SSS	2.2	8.9	0.00	0.19	13.09	11.55	0.24	0.48	0.08	0.00	0.4	262.1
	32SSS	2.5	5.4	0.00	0.00	4.28	0.35	0.03	0.05	0.00	0.00	0.0	26.9
	45SSS	4.0	43.0	0.41	0.00	48.23	14.27	0.99	11.37	2.21	0.41	1.7	3331.8
SSS/IDT (*n* = 2)	24 SSS/IDT	2.7	13.6	0.00	0.15	11.08	3.75	0.95	11.53	2.12	0.14	0.4	406.3
	44SSS/IDT	18.6	58.0	0.00	0.20	69.15	10.68	1.00	0.21	0.04	0.00	3.8	4726.0
Uknown(*n* = 2)	10UN	4.0	21.9	0.00	0.00	10.51	2.57	0.12	5.78	0.73	0.14	0.0	424.7
	23UN	2.7	4.3	0.00	0.00	0.95	0.06	0.00	0.67	0.14	0.04	0.0	7.9
LSD Fisher _(P < 0.05)_ (*n* = 30 inbred lines)		9.379	6.924	0.039	0.03	9.90	5.03	0.331	1.771	0.3632	0.103	8.404	15.705

*^a^Mean ear rot severity is reported as the average of the entry least square means (back-transformed to the original 0-100% disease severity scale)*.

In 2012, ear rot severity positively correlated with fumonisin content in grain samples (FB_1_, FB_2_, and FB_3_), with r factor ranging from *r* = 0.82^***^ to 0.91^***^. The correlations between individual fumonisins were also highly significant (in grain samples they ranged from *r* = 0.90^***^ to *r* = 0.93^***^ *p* < 0.005 and for cobs they ranged from *r* = 0.92^***^ to 0.97^***^ *p* < 0.005). No correlations were found between the ear rot severity and toxin content in maize cobs.

### Molecular Markers and Population Genotyping

**Figure 6:**
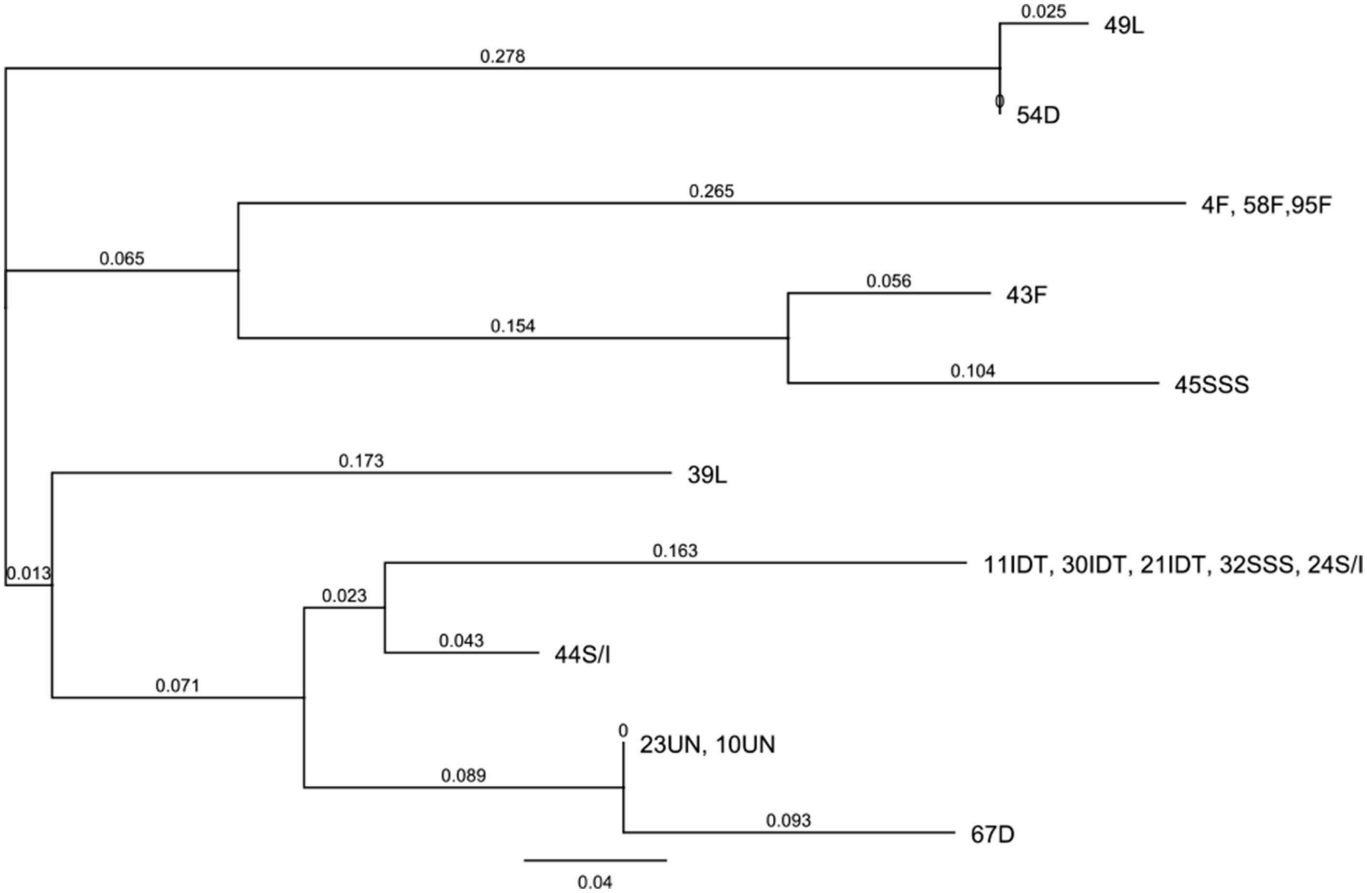
Hierarchical clustering of the inbred lines representing moderate resistant (MR) and susceptible (S) genotypes using Neighbor-joining tree representing phylogenetic distances between objects of relationship matrix.

**Figure 7:**
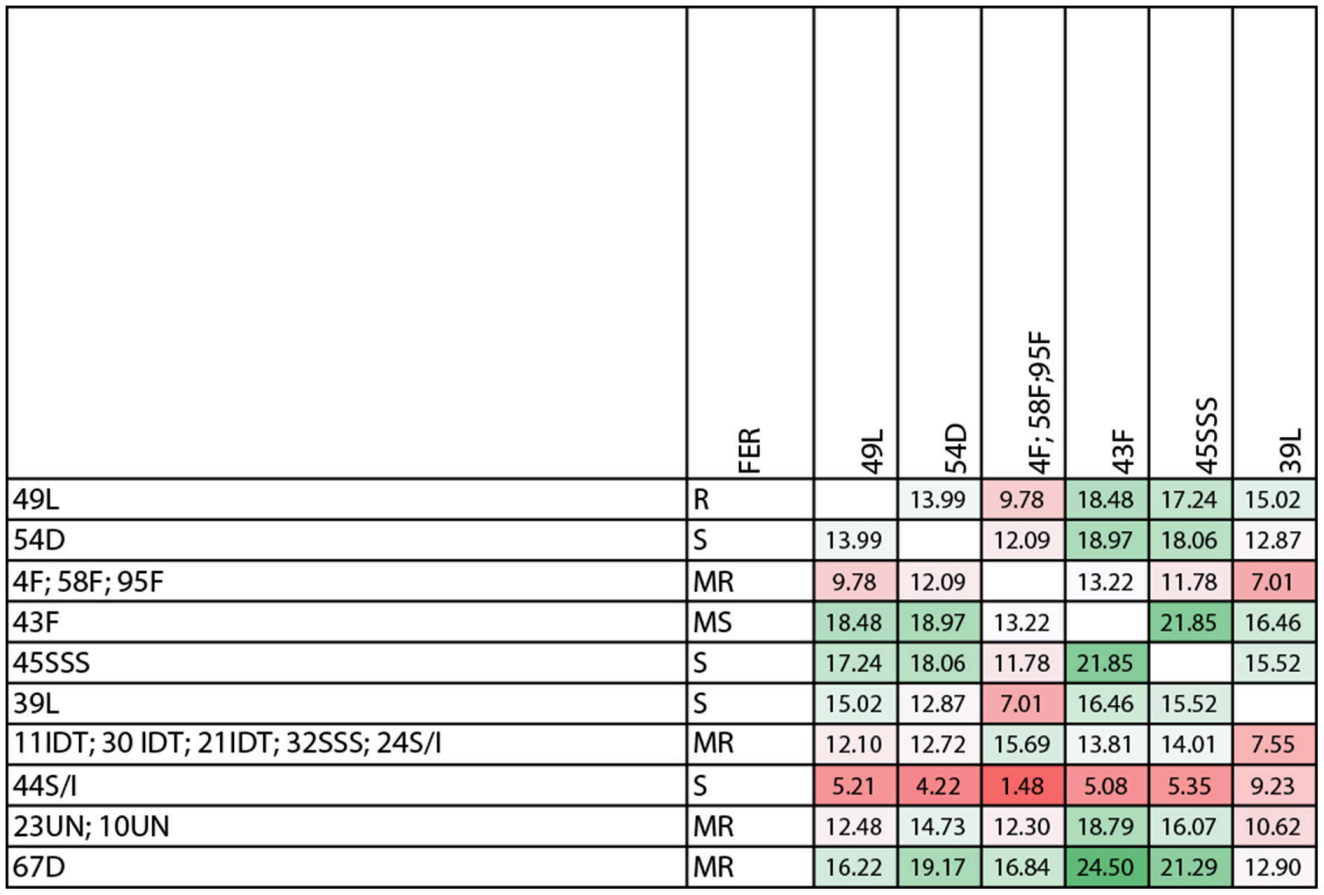
Co-ancestry heat map (the color code corresponds to the degrees of similarity-from green means the most similar to red as the least similar). Inbred lines belong to heterotic group: dent (D), flint (F), Iodent (IDT), Lancaster, Stiff Stalk Synthetic (SSS), SSS/IDT and unknown pedigree (un).

The level of genetic heterogeneity among inbreds with contrasting ear rot resistance was studied using the ddRADseq genome sampling method. They belonged to the following groups: flint, dent, Lancaster, IDT, SSS and SSS/IDT. The range of quality values across all bases at each position for our results in the FastQ file was high and it was conceivable to observe a difference for a number of reads for genotypes, even if they belonged to the same origin group (Figure 6; Supplementary Figures 2, 3). The genotype tree was made using the neighbor-joining method with a genetic distance model. Branch length expressed nucleotide differences. The relative genetic distances between haplotypes were high. One haplotype contained moderately resistant inbred lines belonging to the IDT historical heterotic group (lines 11, 21, 30), SSS / IDT (line 22) and SSS (line 32). Susceptible inbreds belonged to the SSS (line 45) and SSS/IDT groups (line 44) and were classified separately. Likewise, lines from the Lancaster group, described as resistant (line 49) and susceptible (line 39) did not belong to the same haplotype. Furthermore, in the dent group, resistant and susceptible lines were separate. Interestingly, resistant lines from the flint heterotic group belonged to the same haplotype and susceptible flint line was classified separately. Haplotypes of the genotype tree corresponded to the data of the co-ancestry heat map (Figure 7).

## Discussion

The rapid increase of maize cultivation area, the use of inappropriate crop rotation and global warming have resulted in a rise in frequency of diseases including ear rot fusariosis (*Fusarium* spp.). Using highly resistant hybrids is one of the most important methods of integrated plant protection (Vasileiadis et al., 2011; Zijlstra et al., 2011).

Artificial inoculations are necessary for the evaluation of maize FER under field conditions because the disease symptoms under natural infection are not repeatable and largely depend on local environmental factors (Santiago et al., 2015). Different methods of inoculation were developed and described (Drepper and Renfro, 1990; Schaafsma et al., 1993; Munkvold and Carlton, 1997; Munkvold et al., 1997; Reid et al., 2002). These methods correspond with natural disease development processes. Flowering and kernel drying are critical periods for disease development and kernel contamination with fumonisins (Munkvold, 2003b; Bush et al., 2004; De La Campa et al., 2005; Maiorano et al., 2009a,b; Venturini et al., 2011; Cao et al., 2014a,b; Czembor and Matusiak, 2014). Weather conditions affect all of these processes. We could not confirm that high rainfall or high temperature just before harvest are conducive for fumonisin contamination, particularly concerning rainfall data for 2011 and 2012.

In our study, the inoculation technique was very effective, both for FER assessment and for the assessment of the fumonisin contamination of the grain. Kernel samples collected from the most resistant inbred line were also not heavily contaminated with FBs after inoculation. The correlation between FB_1_, FB_2_ and FB_3_ contamination of grain samples and FER severity were highly significant. No significant correlations were observed between the content of fumonisins in samples collected from cobs, both under natural infection and after inoculation. Also in other studies the correlation between the concentration of fumonisin and the severity of FER after inoculation is usually found. Desjardins et al. (2002) and Desjardins and Plattner (2000) reported a very high correlation between fumonisin concentration and visible symptoms, with fumonisin levels being higher in symptomatic kernels and lower in symptomless kernels, respectively. However, in some studies the correlations were not present (Clements et al., 2003a,b).

NGS techniques provide new tools for overcoming the long selection process and increase the breeding efficiency. The knowledge of the QTL genomic location is essential to investigate their potential, as well as their effectiveness toward FER and environmental conditions examined locally (Campos-Bermudez et al., 2013). Here, ddRADseq genome sampling method was deployed to distinguish genetic differences between genotypes with contrasting ear rot resistances (selected based on the field evaluation after inoculation and fumonisin quantification) but also belonging to different heterotic groups: flint and dent groups, as well as to the historical heterotic groups, such as Lancaster, IDT, SSS. The genetic distances between haplotypes were large, indicating an ancient gene pool and possible interspecific hybridization events in maize ancestry. However, among the among the lines of the flint group, as well as in the historical groups, it was possible to find relationship between moderately resistant and susceptible genotypes. Lanubile et al. (2012) summarized reports on mapping disease resistance genes in maize. Using SNPs it was found that QTL located on chromosomes 1, 5, and 9 play significant roles in FER resistance (Zila et al., 2013, 2014). Seven SNPs in six genes associated with FER resistance were identified on chromosomes 4, 5, and 9 based on evaluation of the collection of 1,687 maize inbred lines. Maschietto et al. (2017) found eight QTL associated with FER resistance and FB_1_ contamination and Lanubile et al. (2014) mapped 24 candidate genes for FER resistance on the same chromosomal regions. Based on these findings, it is possible to conclude that the QTL with the highest effect for FER resistance was located distally on Chromosome 1. Wisser et al. (2006) postulated that known disease resistance QTL covered 89% of the maize genome. This high degree of coverage is unlikely and possibly related to the relatively low precision and accuracy of QTL mapping and not taking into account other relevant factors. Our study stands in line with this hypothesis. It is because of the strong influence of environmental factors, plant phenotype and/or interaction of factors, on the spread of the disease in different locations, where FER resistance studies are conducted. That's why QTL mapping results have often been contradictory and their verification in different genetic backgrounds is necessary (Pérez-Brito et al., 2001; Robertson-Hoyt et al., 2006; Ding et al., 2008; Santiago et al., 2013). A new approach to identify candidate genes and QTL for resistance is represented by plant metabolome investigation after pathogen infection (Lanubile et al., 2017) and differential transcriptomic analyses showing genes potentially involved in resistance processes.

Maize breeding has undergone significant changes through the last four decades and so have the materials used. Facing the dramatic climate changes it becomes obvious that pathogen populations are being changed as well, along with the maize lines grown. *Fusarium* ear rot resistance remains one of the most important traits in constant selection of materials and sources of this resistance have never been compared until now. This research has demonstrated that currently grown Polish inbreds and also the ones grown in the past may serve as a valid source of resistance to FER. A strong association was observed between visible *Fusarium* symptoms after inoculation with fumonisin concentration in grain samples, suggesting that selection in maize for reduced visible molds should reduce the risk of mycotoxin contamination. Under natural infection, the cobs contained more fumonisin B_1_ than grain samples. This finding suggests that *F. verticillioides* contaminates grain more intensively when applied artificially. When found naturally, it tends to overgrow preferably the cobs and not the grain. Moreover, under natural infection one has to take into account the presence of other species like *F. graminearum, F. poae, F. temperatum* or *F. proliferatum*, which may compete for nutrients and it has been shown that mycotoxin biosynthesis is one of the strategies used by the pathogen to get the advantage over a competitor. To validate this hypothesis a different experimental approach will be established in the future research. Resistance to FER and to fumonisin accumulation is determined by many factors, such as: environmental conditions, infection timing, presence of other competitive species, ear morphology, the kernel's structure and nutrient components, as well as by the interaction between all these factors, which makes drawing conclusions from a single experimental approach challenging. The use of NGS technique in combination with disease symptom screening and mycotoxin contamination allows for fast analysis which genotypes will be most valuable in incorporating the resistance into current breeding programs.

## Data Availability

This manuscript contains previously unpublished data. The name of the repository and accession number are not available.

## Author Contributions

EC designed and conducted field experiments. AW conducted toxins analyses. MP, UP, and JC performed the molecular studies. EC, AW, MP, and UP interpreted the data. EC, AW, MP, UP, JC, and LS drafted the manuscript, designed figures and tables. All authors read and approved the final manuscript.

### Conflict of Interest Statement

The authors declare that the research was conducted in the absence of any commercial or financial relationships that could be construed as a potential conflict of interest.

## Supplementary Material

Supplementary Figure 1 Seven-point scale used to score FER: 1 = no visible disease symptoms, 2 = 1–3%, 3 = 4–10%, 4 = 11–25%, 5 = 26–50%, 6 = 51–75%, and 7 = 76–100% of kernels exhibiting visual symptoms of infection, such as brown, pink, or reddish discolouration of kernels and pinkish or white mycelial growth (Clements et al., 2003a,b).

Supplementary Figure 2 Number of reads before (blue) and after (red) removing duplicates from data to reduce the impact of a heterogenous PCR step prior to sequencing.

Supplementary Figure 3 The range of quality values across all bases at each position in the FastQ file. The y-axis shows the quality scores. The higher the score, the better the base call. The background of the graph divides the y axis into very good quality calls (green), calls of reasonable quality (orange), and calls of poor quality (red). The elements of the plot are as follows: the yellow box represents the inter-quartile range (25–75%), the upper and lower whiskers represent the 10 and 90% points. The blue line represents the mean quality.

Supplementary Table 1 1. Table of relevant maize accession numbers with indexes and barcodes used, which allow to de-multiplexing NGS resultant sequences. 2. Link to the cloud with raw resultant sequencing data, which is available for everyone after logging to BaseSpace Cloud.
