# Supplementary figures and images for: Differences in Ear Rot Resistance and Fusarium verticillioides-Produced Fumonisin Contamination Between Polish Currently and Historically Used Maize Inbred Lines
Source: Front Microbiol. 2019 Mar 18;10:449. doi: 10.3389/fmicb.2019.00449 (PMC6431649; doi:10.3389/fmicb.2019.00449)

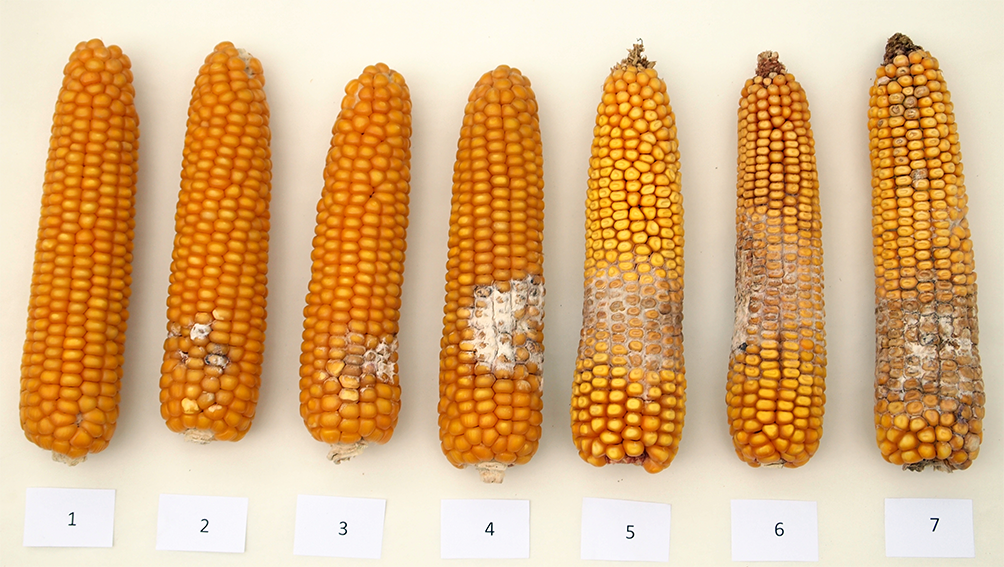

Supplement: Supplementary Figure 1 — Seven-point scale used to score FER: 1 = no visible disease symptoms, 2 = 1–3%, 3 = 4–10%, 4 = 11–25%, 5 = 26–50%, 6 = 51–75%, and 7 = 76–100% of kernels exhibiting visual symptoms of infection, such as brown, pink, or reddish discolouration of kernels and pinkish or white mycelial growth (Clements et al., 2003a,b). [file Image_1.TIF]

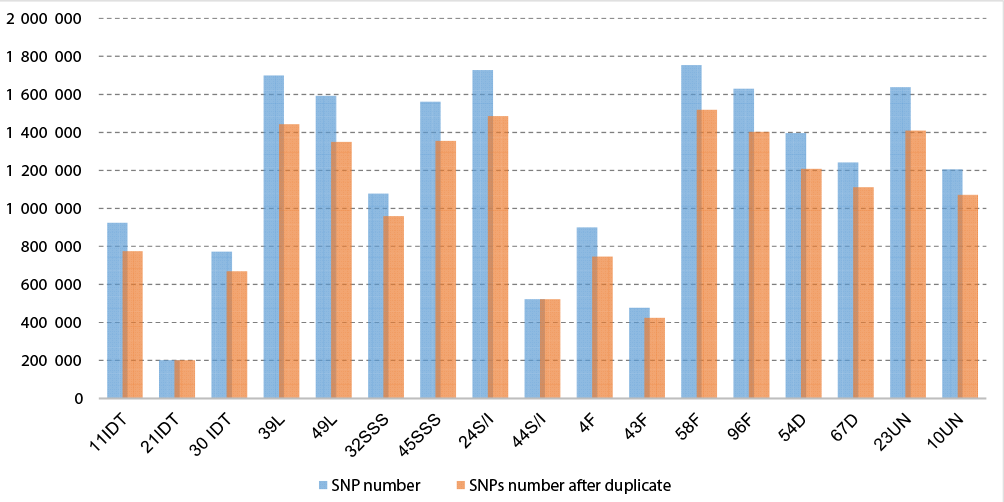

Supplement: Supplementary Figure 2 — Number of reads before (blue) and after (red) removing duplicates from data to reduce the impact of a heterogenous PCR step prior to sequencing. [file Image_2.TIF]

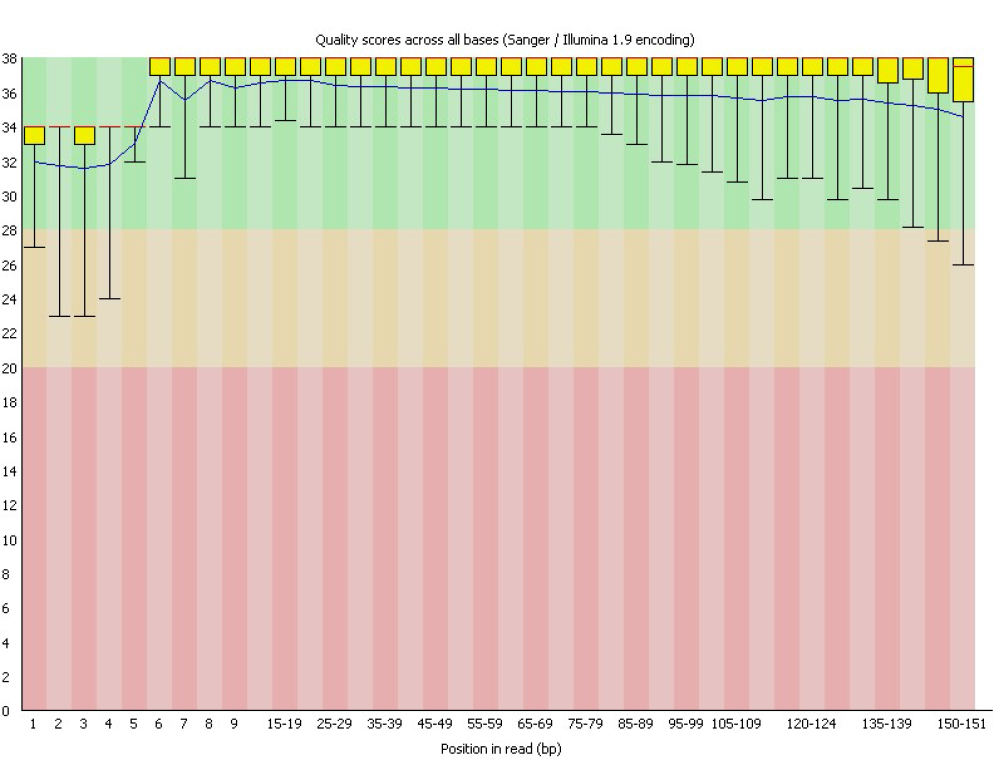

Supplement: Supplementary Figure 3 — The range of quality values across all bases at each position in the FastQ file. The y-axis shows the quality scores. The higher the score, the better the base call. The background of the graph divides the y axis into very good quality calls (green), calls of reasonable quality (orange), and calls of poor quality (red). The elements of the plot are as follows: the yellow box represents the inter-quartile range (25–75%), the upper and lower whiskers represent the 10 and 90% points. The blue line represents the mean quality. [file Image_3.TIF]
